# The Seaside Season

**Published:** 1888-08-18

**Authors:** 


### The Seaside Season.

Once more the holiday season has come round again, and the towns are being deserted for seaside or country resorts. Nor is it only the rich to whom change of air is necessary at this period of the year: overworked servants, underpaid seamstresses, and others to whom the London season has been all work and no play, are now breaking down in health. Alas! their pockets are empty, both occupation and health are gone, and where are they to get the fresh air and rest they need so badly? The physicians of the large hospitals know this class of men and women well; during the hot days of August they haunt the out-patients' department, and beg to be taken into the wards. But the beds there are needed for more serious cases, and applications to the secretary of the Samaritan Fund only draw forth the answer that all the convalescent homes are full, that thousands are waiting their turn to get into them. There certainly are not enough convalescent homes, or not enough that are properly managed. It would be only showing a slight and just interest in their poorer neighbours if a few of those who have houses or lodgings at Brighton, Folkestone, Margate, Bognor, Eastbourne, or other places would pay a visit to the convalescent or holiday homes in the neighbourhood, and find out whether they cannot help the great want for more such homes which undoubtedly exists. And it is no use to go in a prepared state of mind, ready to laud the worthy efforts of the charity, and to exclaim with pleasure at the sight of the tidy dormitories. When a difficulty has to be met, it should be done with a critical mind; an effort should be made to see all from a dispassionate point of view, not from the standpoint of the matron or officer who is showing you round, and who naturally desires to make the best of the institution with which he is connected. There is a book published by the State Charities Aid Association of New York, called a "Handbook for Hospital Visitors," which contains some 150 pages of sound advice to those who are in the habit of inspecting institutions hastily and superficially. We all know what the annual inspection of the committee means in many public buildings. All is put straight for the day, a large lunch is provided, everything disagreeable is put in the background, and only sunny faces are allowed to show. The casual visitor, if he is intelligent, is far more likely to grasp the true state of affairs in a home than a member of the committee is. He will see good points and bad, and either come away prepared to lend a helping hand to such a worthy charity, or else puzzled at the faults he has seen, and eager to learn how they can be overcome. Many cases are inadmissible to a convalescent home because they are just a little too weak or helpless, or have some abscess which will not close. These are just the patients who would most benefit by change of air and good food, and the stern list of ineligibles shuts out many a poor fellow who is pining away for a breath of the invigorating salt sea air. Read through the following rules, and pity those against whom they fulminate:—

Candidates Ineligible.—1. Children under ten years of age. Helpless, dumb, mentally unsound, paralysed, and blind persons. 2. All persons requiring active medical or surgical treatment, or constant watching, nursing, or special diet. Persons suffering from old-standing chronic incurable diseases, mere old age, or constitutional debility. 3. Persons having pulmonary consumption, in any stage of the disease. 4. Persons having cancers, ulcers, or abscesses attended with discharge. 5. Convalescents from small-pox, scarlet fever, cholera, measles, chicken-pox, or typhus. Persons likely, by severe coughing, or their painful appearance, to alarm, disturb the rest, or retard the convalescence of others. 6. Persons subject to epileptic or other fits. 7. Persons of immoral character, conduct, or conversation. 8. Persons troubled with vermin, nits, or catching skin complaints. 9. Persons previously guilty of misconduct in this or any other hospital.

Just the very saddest and most deserving cases are thus precluded from most of the homes. True, it would never do to take in numerous epileptics, dumb people, children, or consumptives; but then they ought to have special homes built for them. If only some millionaire would build a home for all these poor outcasts! But what a large home it would have to be!

